# Mycelium-based biomaterials as smart devices for skin wound healing

**DOI:** 10.3389/fbioe.2023.1225722

**Published:** 2023-08-15

**Authors:** Marco Ruggeri, Dalila Miele, Marco Contardi, Barbara Vigani, Cinzia Boselli, Antonia Icaro Cornaglia, Silvia Rossi, Giulia Suarato, Athanassia Athanassiou, Giuseppina Sandri

**Affiliations:** ^1^ Department of Drug Sciences, University of Pavia, Pavia, Italy; ^2^ Smart Materials, Istituto Italiano di Tecnologia, Genova, Italy; ^3^ Department of Public Health, Experimental and Forensic Medicine, University of Pavia, Pavia, Italy

**Keywords:** mycelium, *Ganoderma lucidum*, *Pleurotus ostreatus*, biocompatibility, gene expression, murine model

## Abstract

**Introduction:** Recently, mycelia of *Ganoderma lucidum* and *Pleurotus ostreatus*, edible fungi, have been characterized *in vitro* as self-growing biomaterials for tissue engineering since they are constituted of interconnected fibrous networks resembling the dermal collagen structure.

**Aim:** This work aims to investigate the biopharmaceutical properties of *G. lucidum* and *P. ostreatus* mycelia to prove their safety and effectiveness in tissue engineering as dermal substitutes.

**Methods:** The mycelial materials were characterized using a multidisciplinary approach, including physicochemical properties (morphology, thermal behavior, surface charge, and isoelectric point). Moreover, preclinical properties such as gene expression and *in vitro* wound healing assay have been evaluated using fibroblasts. Finally, these naturally-grown substrates were applied *in vivo* using a murine burn/excisional wound model.

**Conclusions:** Both *G. lucidum* and *P. ostreatus* mycelia are biocompatible and able to safely and effectively enhance tissue repair *in vivo* in our preclinical model.

## 1 Introduction

Chronic wounds, including venous leg ulcers, arterial ulcers, diabetic ulcers, pressure ulcers, and also bed sores, normally fail to proceed through the normal healing process (Ruggeri et al., 2020). On top of being an economic burden on the healthcare system, these wounds, resulting from different diseases, including tumors and metabolic disorders, infections, and trauma, pose significant health problems (Eming et al., 2021; Lu et al., 2021).

Wound healing is a complex process composed of a series of sequential and overlapped cellular and molecular stages (hemostasis, inflammation, proliferation, and remodeling), that aims to repair damaged tissue and restore skin functions (Yu et al., 2022). In chronic wounds, the healing process is impaired, and the inflammatory stage persists longer than needed, enhanced by the continued secretion of pro-inflammatory mediators (Contardi et al., 2021). This causes delay in epithelialization up to the tissue necrosis, eventually leading to granuloma formation, fistula occurrence, wound dehiscence, permanent ulcers, and excessive bleeding (Andrews et al., 2021). Moreover, the open skin lesions could favor wound colonization and infection, thus further inhibiting a proper wound healing. Moreover, a persistent lesion could increase bacteremia, exposing the patient to the risk of sepsis and, ultimately, to death (Thompson et al., 2019; Bobov et al., 2022).

Currently, traditional wound healing systems present some limitations, involving multiple steps of medication and, eventually, surgical procedures. For these reasons, a long period is required to restore skin integrity and function. Consequently, reducing the healing time and treatment steps could constitute a clear advantage, reducing infection risks, costs, and enhancing the overall patient welfare (Pereira and Bartolo, 2016).

Tissue engineering offers a promising approach to skin repair, aiming to regenerate damaged tissue, using substitutes. In recent years, bio-based functional materials received attention in tissue engineering as green, sustainable, and renewable alternatives to synthetic or non-degradable polymers (Alima et al., 2022). Currently, the use of renewable resources and living systems is tremendously expanding in the field of material sciences, since they are available in nature, affordable, sustainable, and present good physico-chemical properties and promising bioactivities. What is even more appealing are the green production processes of such naturally-derived substances, which can be based on the generation of second raw materials, the valorization of agro-industrial wastes with a preferential use of water-based procedures. Overall, these environmentally friendly approaches do not cause additional harm to the ecosystem, which is now recognized as an essential criterion when designing a new tissue engineering platform. Main advantages of these functional biomaterials are biocompatibility, low immunogenicity, acceptable biodegradation rate, and a 3D structure similar to the natural extracellular matrix (ECM), able to actively promote cell response (Suarato et al., 2018; Narayanan et al., 2020; Manan et al., 2022).

In this context, mycelia materials are rapidly gaining interest in the biomedical field. Mycelium is the self-growing vegetative part of filamentous fungi and has been recently proposed as a potential novel platform for wound healing due to its interwoven three-dimensional, interconnected fibrous networks similar to the dermal collagen structure (Antinori et al., 2021). This network is made of hyphae, which grow in a thread-like architecture, resembling the dermal extracellular matrix (Haneef et al., 2017; Sayfutdinova et al., 2022). The filamentous structures, the mechanical strength, and the presence of bioactive molecules such as polysaccharides, phenols, polyphenols, and carotenoids in the mycelia of edible fungal species offer optimal features to develop smart and green scaffolds for tissue engineering, with potential anti-inflammatory and antioxidant properties. In addition, being living materials able to sense the surroundings and feed on various substrates, mycelia can acquire particular features depending on their edaphic conditions, thus tuning their mechanical properties and their overall morphology, and becoming elastic, porous, stiff (Haneef et al., 2017; Sayfutdinova et al., 2022; Gantenbein et al., 2023; Peng et al., 2023). Interestingly, these biocompatible and biodegradable mycelium-based materials can be directly obtained via culture in a water-like environment (culture broth), without the need of toxic solvents, nor the use of cumbersome fabrication techniques or post-growth processing, thus constituting a real green alternative in the biomedical materials field. Mycelia, both alone and in combination with other materials, find applications in a plethora of different areas, including cosmetics, biosensors, and smart packaging (Vandelook et al., 2021; Gantenbein et al., 2023).

Given these premises, this work aims to investigate the biopharmaceutical properties of fibrous structured mycelia of *Ganoderma lucidum* and *Pleurotus ostreatus* (edible fungi) to prove their safety and effectiveness in tissue engineering as dermal substitutes. The mycelial materials were autoclaved after growth and characterized using a multidisciplinary approach, including physicochemical features (morphology and elemental composition, thermal behavior, and surface charge and isoelectric point). Moreover, preclinical properties such as gene expression and *in vitro* wound healing assay have been evaluated using fibroblasts. Finally, the *in vivo* safety and efficacy of these naturally-derived substrates were assessed using a murine burn/excisional wound model.

## 2 Materials and methods

### 2.1 Strain, media, and growth conditions

Active cultures of *G. lucidum* (DSM9621) and *P. ostreatus* (DSM11191) were purchased from DSMZ (Germany) and maintained in a 100 mm Petri dish with Potato Dextrose Broth (PDB, Merck) as the growth medium. The growth medium was changed to fresh every 30 days. A piece of 20-day-grown mycelium was inoculated in 100 mm Petri dishes containing 30 mL of PDB at 24 g/L in water. All the media were autoclaved before use (120°C for 20 min) via SYSTEC-VX 40. The grown mycelia were incubated in a climatic chamber (Memmert, HPP 260), at 27°C and 78% relative humidity, in the dark.

### 2.2 Mycelia scaffolds preparation

After 20 days of growth (surface of the Petri dish totally covered), the mycelium was collected and cleaned from the PDB growth substrate by means of a spatula and deionized water washing. Subsequently, the materials were autoclaved at 120°C for 20 min, then dried under a laminar hood and further irradiated for 10 min in a distance of 15 cm with a commercial 254 nm UV germicidal lamp (30 W) to dry the mycelia and to preserve sterlity. This protocol has been previously established by Antinori et al., as both strains appear inactive after growth treatment while keeping the cell wall intact.

### 2.3 Morphological characterization

In order to investigate the surface morphology, mycelia were fixed in a solution of 2% v/v glutaraldehyde in 0.1 M cacodylate buffer for 2 h at room temperature. After a final washing in MilliQ water, the mycelia were dehydrated with the following procedure: a series of 10-min incubations in solutions of increasing ethanol concentrations in water (from 30% to 100% v/v), subsequently incubation in 1:1 ethanol:hexamethyldisilazane (HMDS, Sigma-Aldrich), then a final immersion in 100% HMDS, and overnight drying in air. Finally, the samples were sputtered with a 10 nm gold layer to be analyzed with a JEOL JSM-6490LA Scanning Electron Microscope (SEM) equipped with a tungsten filament and operating at an accelerating voltage of 10 KV.

A Transmission Electron Microscope (TEM) analysis was conducted to investigate the internal mycelial structure. Briefly, samples were fixed with glutaraldehyde as described above, post-fixed in 1% osmium tetroxide in MilliQ water for 2 h, and then stained with an aqueous 0.5% uranyl acetate solution overnight at 4°C. After several washes in MilliQ, dehydration of the samples was performed in a series of ethanolic solutions, before embedding the material in Spurr resin (Sigma Aldrich, Italy). Sections of about 70 nm were obtained with a diamond knife on a Leica EM UC6 ultramicrotome and observed under a Jeol JEM 1011 (Jeol, Japan) electron microscope equipped with a 2 Mp charge-coupled device camera (Gatan Orius).

### 2.4 Thermogravimetric analysis

A thermogravimetric analysis (TGA) was performed using a TGA Q500 (TA Instruments) to assess the mass loss and nature of the species evolved during thermal decomposition, as well as the thermal stability of mycelia. Measurements were performed on 5–8 mg samples in an aluminum pan under an inert N_2_ atmosphere, with a flow rate of 50 mL/min, over a temperature range of 30°C–600°C, with a 5°C/min heating rate. The weight loss and its derivative were recorded simultaneously as a function of time/temperature.

### 2.5 Surface zeta potential

The surface zeta potential (ζ) of the mycelium-based scaffolds was determined by means of an electrokinetic analyzer for solid surface (SurPASS™ 3, Anton Paar GmbH) using a cylindrical cell. The samples (10 × 10 mm^2^) were mounted between two filter disks in the sample holder of the cylindrical cell. A KCl aqueous solution at a concentration of 0.01 mol/L was used as the streaming solvent, and the pH was scanned in the range from 2 to 9 (Ruggeri et al., 2022a).

### 2.6 Biopharmaceutical characterizations

#### 2.6.1 Metabolic activity of fibroblasts

Normal human dermal fibroblasts (NHDF from juvenile foreskin, PromoCell, WVR, Italy) were used as a cellular model to investigate the biocompatibility of the mycelial scaffolds, following ISO 10993-5. The cells were cultured in DMEM supplemented with 200 IU/mL penicillin/0.2 mg/mL streptomycin (Sigma-Aldrich, Italy) and with 10% v/v fetal bovine serum (FBS, Euroclone, Italy) in a humidified incubator at 37°C and with 5% CO_2_. Extracts of *P. ostreatus* and *G. lucidum* samples were prepared as follows. Briefly, 20 mg of autoclaved mycelia were irradiated for 20 min (10 min per side), then immersed in sterile potassium phosphate buffer (PBS, pH 7.4, Gibco), and incubated at 37°C for 24 h. The washing step was repeated a second time (with fresh PBS and additional 24 h of incubation) to ensure a total removal of the PDB. Afterwards, each 20-mg mycelium piece was incubated with 1 mL of a serum-free DMEM for 24 h, and the resulting stock solution (extracted medium) was used to prepare the tested dilutions (1:20, 1:200). These dilutions were selected from the results of an experimental screening previously conducted in our laboratory (Antinori et al., 2021).

Afterwards, NHDFs were seeded in 96-well plates at a density of 3.5*10^4^ cells/well and let attach overnight. The extracted media were then added, and the cells re-incubated for 24 h. After 24 h, a MTT assay was conducted to determine metabolic activity as the ratio between the absorbance read after the contact with the sample and the absorbance read in control (standard growth conditions). Briefly, all samples were incubated with a MTT solution (1 mg/mL) in DMEM without phenol red (Sigma-Aldrich, Milan, Italy) and, after 3 h, absorbance readings at 570 nm (with reference λ = 690 nm) were recorded.

#### 2.6.2 Quantitative PCR

Twenty mg of each scaffold were sterilized and immersed in sterile PBS to remove the excess of PDB, as described above. Afterwards, mycelia were incubated in a serum-free DMEM for 24 h to produce extracted media of different concentrations (0.05 and 0.005 mg/mL). NHDFs were plated at a 2.8 × 10^5^ cells/well density in 12-well plates. When the cultures reached confluency, the mycelia extracted media were added, and the fibroblasts were re-incubated for 7 days. NHDFs were washed, collected and total RNAs were isolated with TriZol agent (ThermoFisher, CA, United States) according to the manufacturer’s instructions. Total RNAs were quantified by using OmegaStarFluo at 230 nm. cDNA was produced using 1 µg of total RNA. Reverse transcription was carried out using iScript™ cDNA Synthesis Kit (Bio-Rad, CA, United States) according to the manufacturer’s instructions. Expression of the BCL-2, and COL-1a coding RNAs were analyzed by quantitative RT-qPCR using SsoAdvanced Universal SYBR Green Supermix (Bio-Rad, CA, United States) and specific primer sets at a final concentration of 400 nM, for 50 ng of cDNA. GADPH expression was used for normalization of the RT-qPCR data. Probe and primers were employed in a concentration of 250 nM. The reverse and forward primers sequences are a Bio-Rad patent.

As indicated by the kit, thermal cycling program was set as follows: polymerase activation was achieved in 30 s at 95°C; DNA denaturation at 95°C for 15 s and annealing at 60°C for 30 s repeating cycles 39 times. Finally, melt curves were recorded.

#### 2.6.3 *In vitro* wound healing assay

Cell motility was studied by means of a wound healing assay: for this purpose, NHDFs were seeded in culture insert 2 well in a 24-well plate designed for fluorescent microscopy (Ibidi). The insert in the wells is formed of 2 chambers with a growth area of 0.22 cm^2^ divided by a septum with a width of cell free gap of 500 μm ± 50 μm. Fibroblasts were seeded in each chamber at 10^5^ cells/cm^2^ concentration and grown at confluence in standard conditions. After 24 h cells reach confluence and the insert was removed displaying 2 areas of cell substrates divided by the gap.

Cell substrates were washed with PBS and incubated with 600 µL of mycelia extracted media (1:200) according to the MTT results and following ISO 10993-5. Cells treated with 600 µL of DMEM were used as positive control. At prefixed timepoints (0, 1, 2 and 3 days), scanning of the wells was performed using Confocal Laser Scanning Fluorescence Microscopy (CLSM). For this purpose, NDHFs were washed three times and fixed with a 4% v/v paraformaldehyde solution in PBS for 1 h. Cells nuclei and cytoskeletons were stained using Hoechst 33,258 (200 μL at 1:10.000 dilution in PBS per each well) and FITC Atto 488 phalloidin (100 μL at 20 μg/mL in PBS in each well), respectively, and observed using CLSM (Leica TCS SP2, Leica Microsystems, Milan, Italy) at λ_ex_ 346 nm and λ_em_ 460 nm for Hoechst 33,258, and λ_ex_ 501 nm and λ_em_ 523 nm for FITC-phalloidin. At the prefixed timepoints, wound reduction area was evaluated using ImageJ software (Schneider et al., 2012). The area covered by the cells overtime was measured by applying thresholding method and the percentage of wound closure was calculated according to Eq. 1, where WA_0_ stands for “wound area at time 0” and WA_t_ is “wound area at prefixed timepoints” (García-Villén et al., 2020):

$$\%\;Wound\;closure=100-\left({WA}_{t}/\;{WA}_{0}*100\right)$$ (1)

### 2.7 Evaluation of systems efficacy on *in vivo* excisional/burn murine model

All animal experiments were carried out in full compliance with the standard international ethical guidelines (European Communities Council Directive 86/609/EEC) approved by Italian Health Ministry (D.L. 116/92). The study protocol was approved by the Local Institutional Ethics Committee of the University of Pavia for the use of animals and by ISS (Istituto Superiore di Sanità). Six male rats (Wistar 200–250 g, Envigo RMS S. r.l.) were anesthetized with equitensine at 3 mL/kg (39 mM pentobarbital, 256 mM chloral hydrate, 86 mM MgSO_4_, 10% v/v ethanol, and 39.6% v/v propylene glycol) and shaved to remove all hair from their backs for the following evaluations (Ruggeri et al., 2022b).

Three circular full-thickness burns, 4 mm in diameter, were produced on the back of the animals by contact with an aluminum rod (105°C for 40 s). After 24 h, the formed blisters were removed using a 4 mm diameter biopsy punch to obtain full-thickness lesions. Mycelia having 4 mm diameter were applied and wetted with 20 μL of saline solution (0.9 g/L). Lesions treated with 20 μL of saline solution served as negative control. Lesions were covered with a sterile gauze, and the rats back was wrapped with a surgery stretch (Safety, Italy) to protect the lesions. At prefixed times after blister removal (0, 3, 6, 10, 13, and 18 days) photographs of the lesions were taken by using a digital camera (Sigma SD 14) which allowed for sizing the lesions and monitoring the healing process. The size of the wounded areas was determined with an image analysis software (ImageJ, ICY, Institute Pasteur, France). Six, 13, and 18 days after the treatment, full-thickness biopsies were taken in correspondence with the initial lesions, and the histological analysis of the excised tissues was performed. A biopsy of intact skin was also taken for comparison.

Tissue samples were bisected along the wildest line of the wound, immediately immersed in the fixative solution (10% neutral buffered formalin), embedded in paraffin and sectioned at a thickness of 5 μm. Some sections were stained with hematoxylin and eosin (H&E), others with picrosirius red (PSR) and observed with a light microscope Carl Zeiss Axiophot.

## 3 Results and discussion

### 3.1 Morphological characterization

**FIGURE 1:**
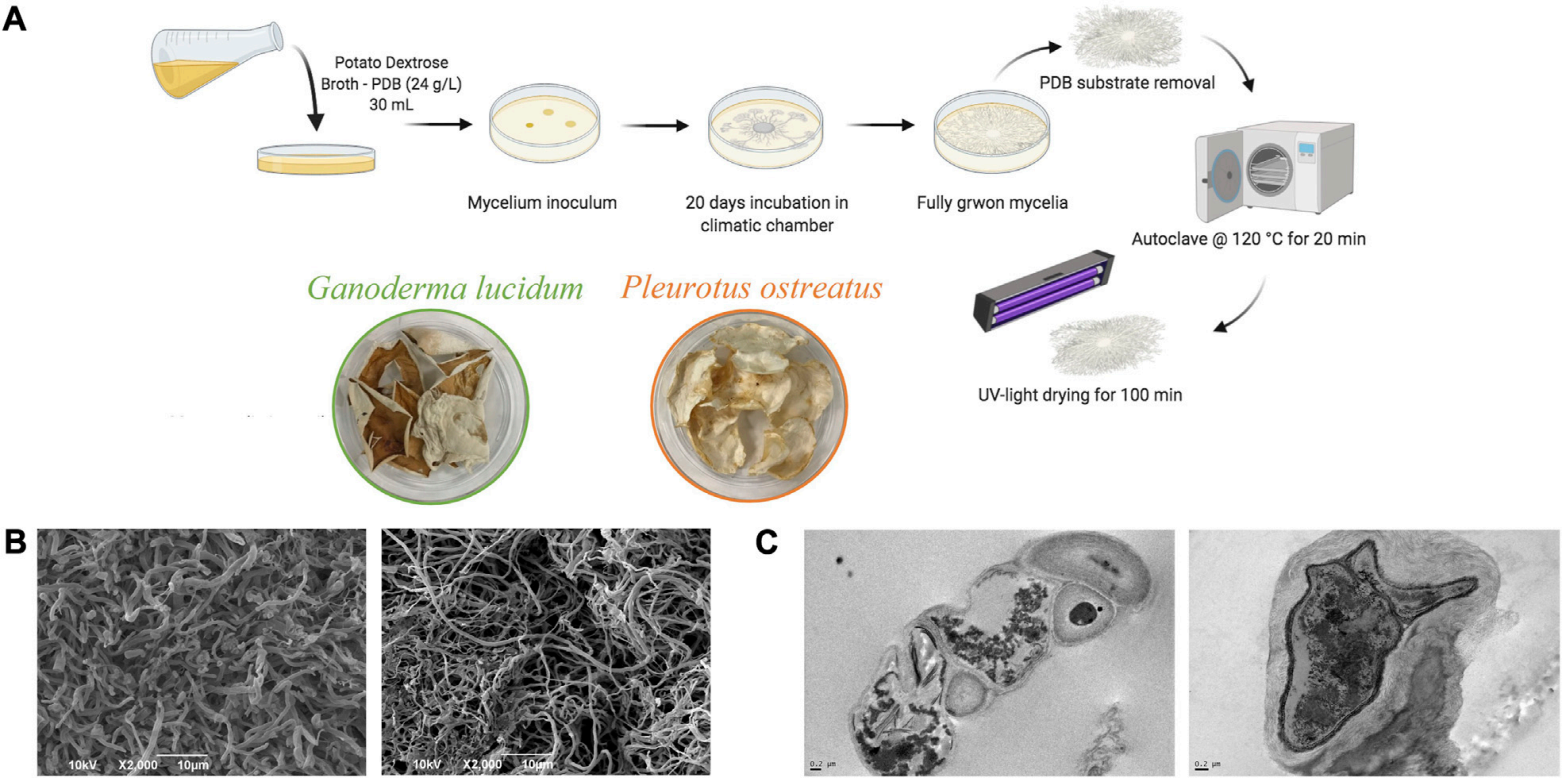
**(A)** Schematic representation of mycelium growth and their scaffolds preparation; **(B)** SEM and **(C)** TEM images of autoclaved mycelia (*G. lucidum* on the left panel and *P. ostreatus* on the right panel).

The fungal mycelia were grown with potato dextrose broth (PDB) in a climatic chamber (27°C, 78% R.H.) until a piece of 20 days-grown mycelium completely covered the surface of a 100 mm Petri dish, to be then collected and leaned from the substrate (Figure 1A). After the harvest, the inactivation of the biological activity of the mycelium was achieved by steam sterilization since it has been previously demonstrated that this treatment led to mycelium complete inactivation (Antinori et al., 2021).

Figures 1B,C report the SEM and TEM images of the mycelia. Both mycelia show a porous structure and a micro-structure characterized by tubular hyphae preserved after steam sterilization. In particular, *G. lucidum* shows a denser structure than *P. ostreatus,* as previously observed, and such differences are attributable to the two different species. However, microfibers in both mycelia are consistent with the intended use as scaffolds for wound healing application. In fact, nano- and microfibers have been proposed as ideal scaffolds due to their similarity to the extracellular matrix made of a fiber network of polysaccharides and proteins. In addition, these structures offer efficient performance for improving the healing process due to the large specific surface area and high porosity with small pore size, as suggested in the literature (Azimi et al., 2020).

TEM analysis proves that steam sterilization does not cause damage to the cell walls, and the hyphae (mainly made of chitin, glucans, and glycoproteins) retain their features (Vandelook et al., 2021), while the internal structure and organelles collapse.

### 3.2 Physico-chemical characterization

**FIGURE 2:**
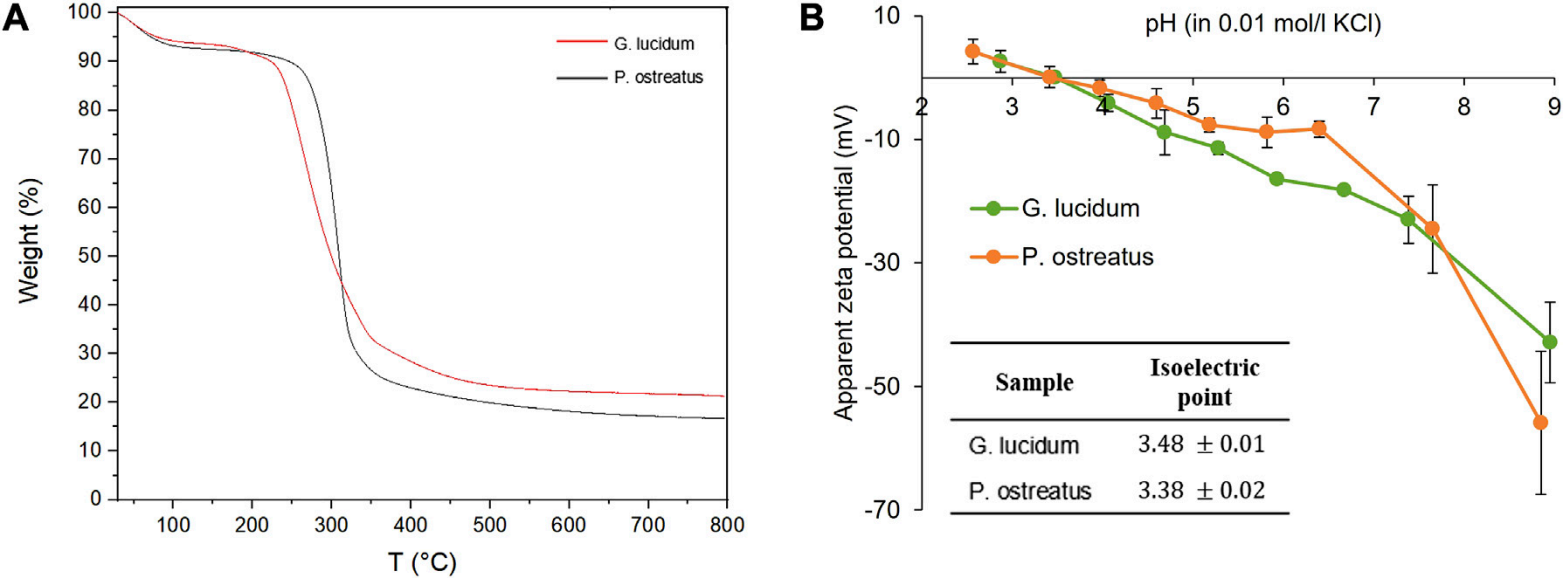
**(A)** Thermogravimetric analysis of *G. lucidum* and *P. ostreatus*; **(B)** Apparent zeta potential (mV) vs. pH and isoelectric points of *G. lucidum* and *P. ostreatus* (mean values ± sd; n = 3).

The thermal degradation of *G. lucidum* and *P. ostreatus* is reported in Figure 2A. Both mycelia are characterized by weight loss in two stages. *G. lucidum* and *P. ostreatus* thermal degradation profiles show a first weight loss at around 90°C, followed by an intense weight loss at around 250°C or 300°C, for *G. lucidum* and *P. ostreatus,* respectively. The first weight loss is conceivably related to the water evaporation, even though in the literature it has been related to the degradation of small amphiphilic proteins, the hydrophobins (Chulikavit et al., 2022). However, in our case, the TGA analysis was performed after authoclaving the mycelia, a process that could have already caused the degradation of those cysteine-rich proteins. The second degradation stage is attributable to the degradation of cell wall polysaccharides and proteins, which constitute the main skeleton of the hyphae. In general, the degradation of *G. lucidum* shows a earlier degradation compared to *P. ostreatus*. These differences could be related to the chitin content. In fact, in our previous work, FTIR analysis revealed stronger amide absorption bands in *P. ostreatus* spectrum, related to a higher chitin content (Antinori et al., 2021).

Figure 2B reports the apparent surface zeta potential profiles of *G. lucidum* and *P. ostreatus* and their corresponding isoelectric points (i_ep_) (in the inset). The evolutions of the apparent ζ with the pH show similar trends for *G. lucidum* and *P. ostreatus* and the derived i_ep_ are 3.48 ± 0.1 and 3.38 ± 0.2, respectively, indicating that the surface charges of the two mycelia are similar. Moreover, both mycelia are negatively charged at physiological pH (−23 mV for *G. lucidum* and −24 mV for *P. ostreatus*). Surface charges and i_ep_ are crucial parameters in cell adhesion and proliferation since they influence the type, quantity, and degree of adsorbed proteins, which in turn affects the establishment of focal adhesions, integrin bonds, and cell adhesion (Kaniuk et al., 2021). Moreover, in a previous work of ours, *P. ostreatus* demonstrates a hydrophilicity greater than *G. lucidum* mycelium: the differences in chemical composition and surface properties could promote better cell proliferation as more hydrophilic surfaces increase cell attachment (Antinori et al., 2021).

### 3.3 Fibroblasts metabolic activity and gene expression

**FIGURE 3:**
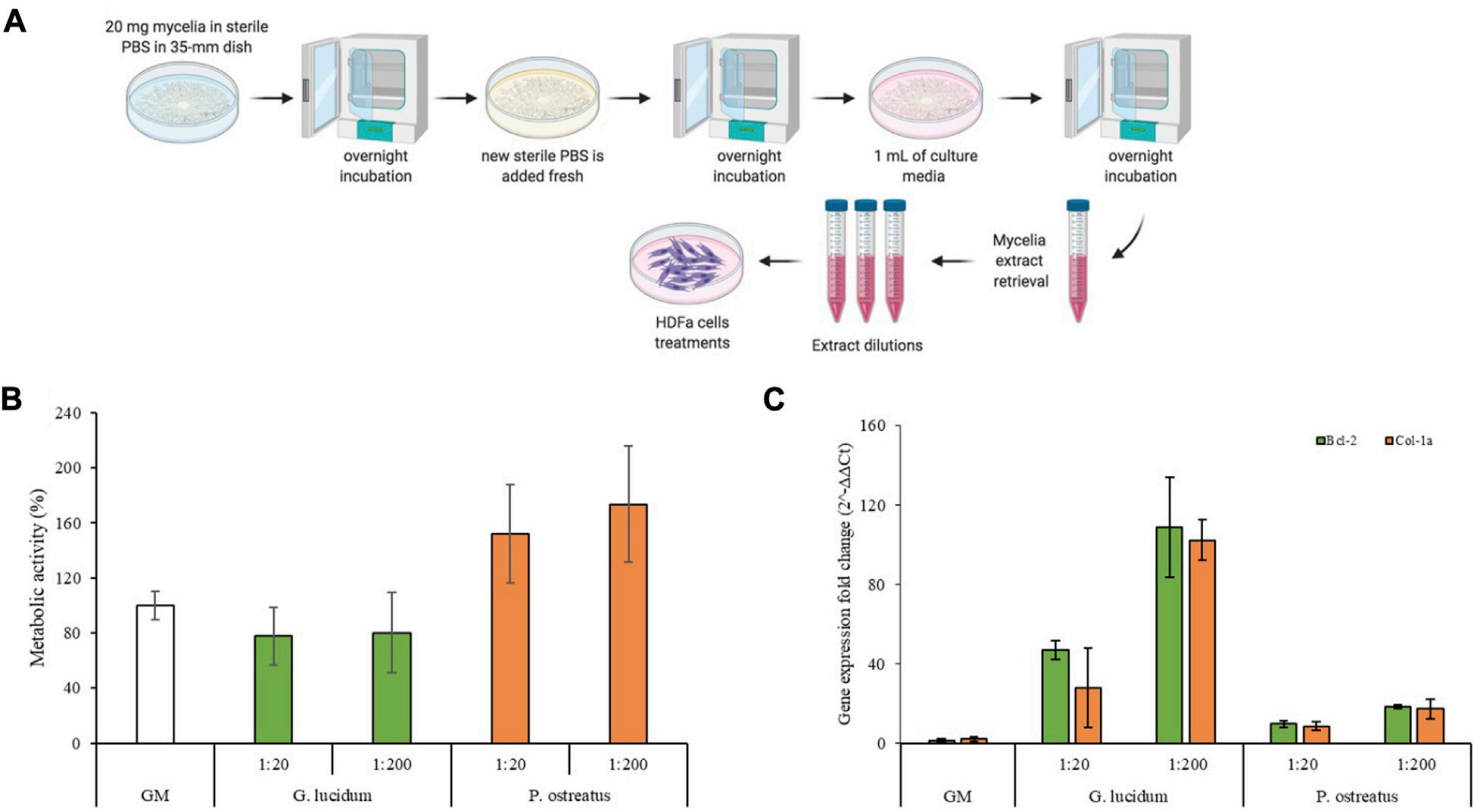
**(A)** Schematic representation of the mycelia extracts preparation; **(B)** Cells metabolic activity of *G. lucidum* and *P. ostreatus* scaffolds (mean values ± sd; n = 6); **(C)** Bcl-2 and Col-1a expression of fibroblasts after 7 days of contact with the mycelia extracts (mean values ± sd; n = 3). ANOVA one-way; Scheffe’s test (*p* < 0.05)—Cell viability: GM vs. *P. ostreatus* 1:20 and *P. ostreatus* 1:200; *G. lucidum* 1:20 vs. *P. ostreatus* 1:20 and *P. ostreatus* 1:200; *G. lucidum* 1:200 vs. *P. ostreatus* 1:20 and *P. ostreatus* 1:200. Bcl-2: GM vs. *G. lucidum* 1:200; *G. lucidum* 1:20 vs. *G. lucidum* 1:200; *G. lucidum* 1:200 vs. *P. ostreatus* 1:20 and *P. ostreatus* 1:200. Col-1a: GM vs. *G. lucidum* 1:200; *G. lucidum* 1:20 vs. *G. lucidum* 1:200; *G. lucidum* 1:200 vs. *P. ostreatus* 1:20 and *P. ostreatus* 1:200.

Cytotoxicity is a key factor in understanding the positive or negative effect that a material has when it comes in contact with cells, on the basis of what is recommended by ISO 10993-5-2009. Cell metabolic activity was evaluated by means of an MTT assay on mycelial extracts, as schemed in Figure 3A. Figure 3B reports the fibroblast viability (%) after 24 h of incubation with mycelia extracts. The results suggest a different activity profile, although both mycelia show excellent biocompatibility. In particular, *G. lucidum* is characterized by good biocompatibility (metabolic activity not statistically different with respect to the positive control), while *P. ostreatus* mycelium significantly increases cell metabolic activity with a value higher than that of the positive control, which agrees with the literature (Taofiq et al., 2019; Antinori et al., 2021).

Moreover, the gene expression was characterized for the tested samples, and Col-1a, encoding the major component of collagen type I, the main part of the extracellular matrix (Scharffetter et al., 1989), and Bcl-2, encoding an anti-apoptotic factor (Singh et al., 2019), levels were measured. Figure 3C reports Col-1a and Bcl-2 gene fold expression of fibroblast cells grown for 7 days in contact with the extraction media of *G. lucidum* and *P. ostreatus*. Quantitative RT-qPCR analysis indicated that both genes are expressed at higher levels in fibroblasts treated with *G. lucidum* and *P. ostreatus* extracts at both concentrations with respect to the control. In detail, *G. lucidum* at concentration of 5 μg/mL (1:200 dilution) exhibits the best performance and significant differences (*p* < 0.05) with respect to the control, with an increase in the expression levels of about 50 and 100-folds for Bcl-2 and Col-1a genes, respectively. This could be related to the β-glucans content of mycelia which plays a crucial role in promoting the synthesis of types I and III collagen, enhancing the wound healing process (Kwon et al., 2009). Interestingly, the expression of Bcl-2 and Col-1a is inversely proportional to the biocompatibility results: the lower their cell viability, the more intense the gene expression. It has been commonly acknowledged that apoptosis owing to external stress stimuli can be rescued by Bcl-2 (Chawla-Sarkar et al., 2004; Ryu et al., 2007; Ardehali et al., 2011). Considering this, it is conceivable to state that the overexpression of Bcl-2 in cells treated with *G. lucidum* represent a survival strategy to tackle the external-induced stress. Contrarily, the homeostasis was not impaired when cells were kept in contact with *P. ostreatus* and no anti-apoptotic overexpression occurred. This is also confirmed by the lower expression of collagen as cells in the proliferative phase are less prone to produce extracellular proteins (Mathew-Steiner et al., 2021).

### 3.4 *In vitro* wound healing assay

**FIGURE 4:**
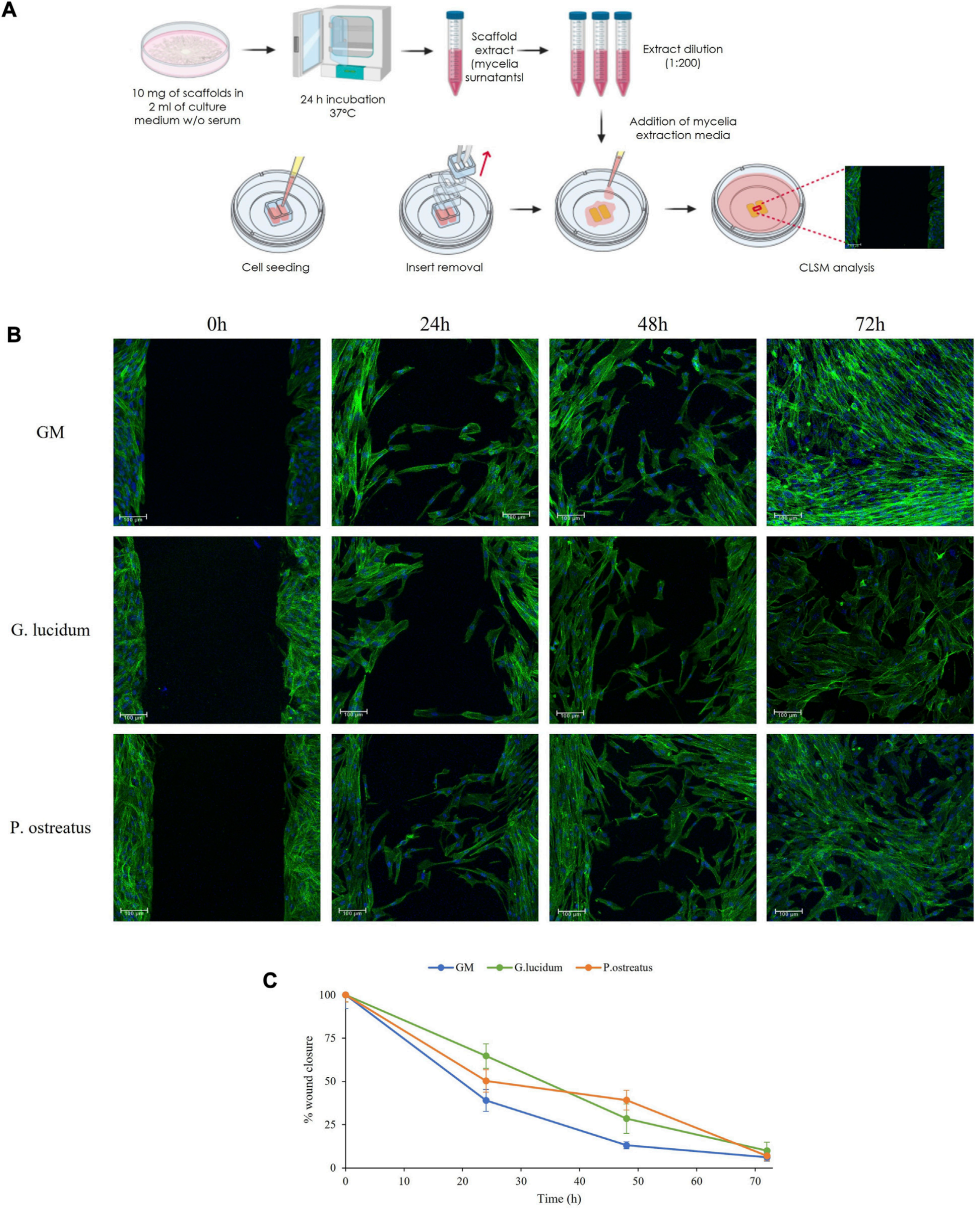
The schematic of the experimental method **(A)**, CLSM images of fibroblasts during the wound healing assay **(B)** and the percentage of gap reduction after 0, 24, 48 and 72 h (mean values ± sd; n = 3) **(C)**.

Figure 4 reports (a) the schematic of the experimental method, (b) CLSM images of fibroblasts during the wound healing assay and (c) the percentage of gap reduction after 0, 24, 48 and 72 h. Mycelia extracts were tested at 5 μg/mL (1:200 dilution) based on the cytotoxicity and PCR-RT results.

At the initial time point, the images show defined gaps of 500 µm (insert dimensions) resembling wounds, and the confluent cells border the gaps. After 24 h and 48 h, fibroblasts start to migrate and form bridges with the cells in the opposite cell substrates crossing the gaps with the typical morphology of migrating cells. After 72 h, fibroblasts close the gap area forming anastomosis, and confluence is achieved in all samples.

Compared to the control, both *G. lucidum* and *P. ostreatus* extracts are characterized by superimposable healing properties. These observations agree with the percentage of wound reduction, where no significant differences are evident, confirming the biocompatibility of both mycelia extracts. Despite viability and semiquantitative RT-qPCR results displayed differences between the two fungal extracts, wound healing CLSM images revealed that, independently from the samples, fibroblasts maintain their morphology during the whole experiment, showing a typical fusiform feature and elongated cytoskeletons.

Previous works have claimed that the wound-healing properties of mushrooms-derived materials have been associated to their rich content of polysaccharides, such as β-glucans, which are able to stimulate cells, and cytokines and growth factors release. In the study of De Jesus *et al.*, the wound healing capacity of β-glucan from *Piptoporus betulinus* was proved on *in vitro* wound healing assay using intestinal cells (de Jesus et al., 2018). In another study, β-glucans derived from *Schizophyllum commune* promoted wound closure by activation of integrin/FAK/Src signaling pathway (Seo et al., 2019). Moreover, it has been also reported that β-glucan obtained from *G. lucidum* was able to induce cytokines suppression: this enhanced cell proliferation and decreased apoptosis, promoting wound reparation (Gao et al., 2002).

### 3.5 *In vivo* wound healing

**FIGURE 5:**
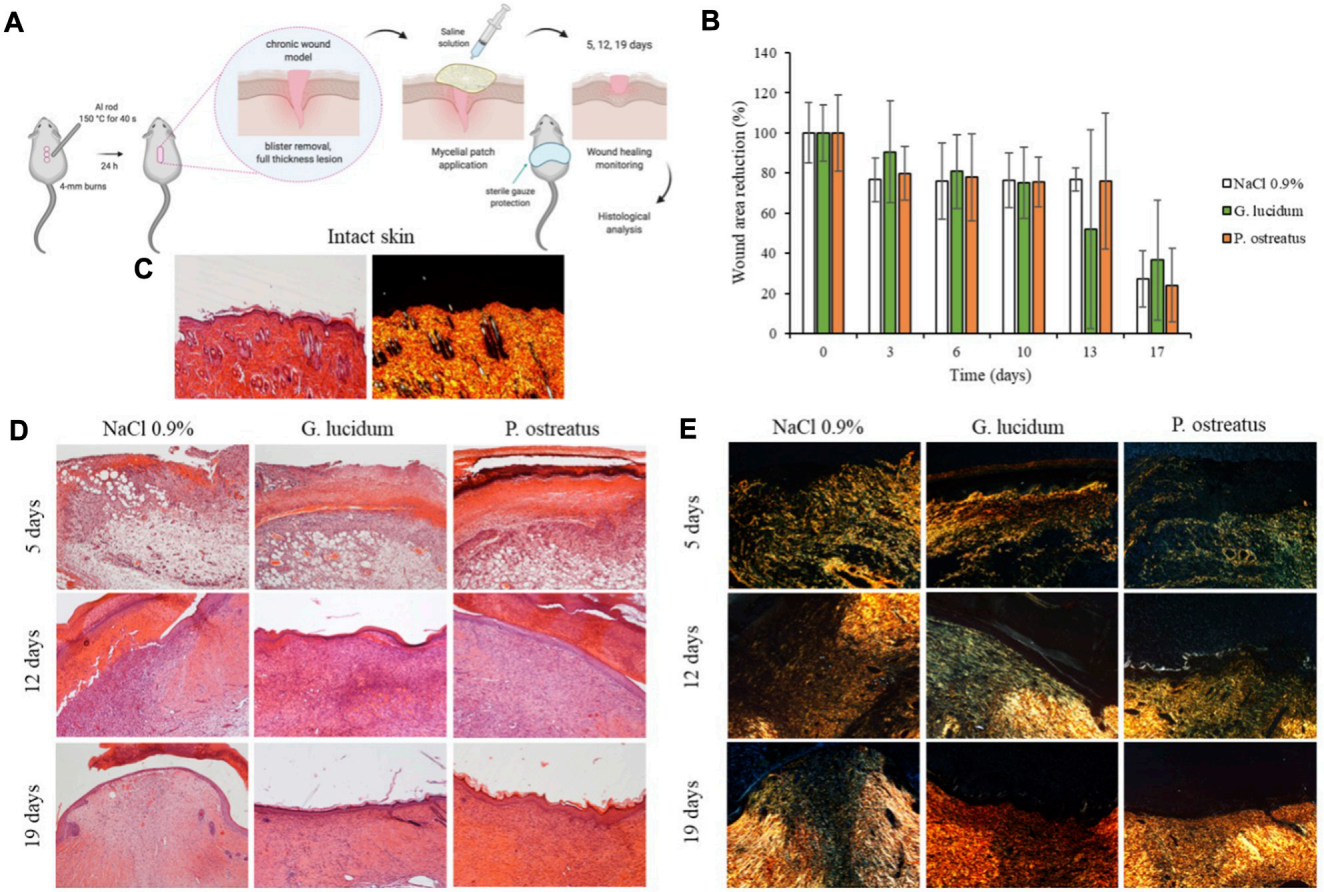
**(A)** *In vivo* wound healing model (murine burn/excisional model); **(B)** Wound area reduction vs. time obtained during the treatments (mean values ± sd; n = 3); **(C)** H&E and PSR of intact skin; **(D)** H&E and **(E)** PSR of lesions treated with *G. lucidum*, *P. ostreatus* and with saline solution as negative control after 5, 12, and 19 days of treatment. Original magnification: 5X. Each micrograph frame has a width of 1780 μm.

To study the wound healing properties of mycelia scaffolds, a murine burn/excisional model was used and the histology of the tissues in correspondence of the implants of *G. lucidum* and *P. ostreatus* has been reported in comparison to the control lesion treated with saline solution and the intact skin (Figure 5). The histological evaluation was performed on rats at 6, 13, and 18 days post-wounding.

The histological examination of wounded tissue on different days of treatment revealed an evident performance of the mycelia in the healing of skin injury. The most significant effects of *G. lucidum* and *P. ostreatus* include stimulation of re-epithelization and accelerated collagen deposition. After 5 days of treatment all samples show typical signs of the proliferative phase of healing process (Singer and Clark, 1999) with the formation of granulation tissue, a mixture of proliferating capillaries, fibroblasts, and inflammatory cells in a loose edematous extracellular matrix. Many neo-formed capillaries (angiogenesis) are evident both in treated samples and in saline control, and the surface of all the tissues is covered with blood clot. PSR stain under a polarization microscope indicates that almost all of the wound area contain green color (rich in type III) collagen, newly deposited witin immature granulation tissue, while red/orange (rich in type I collagen) fibers are rare, despite a few more in the samples treated with *P. ostreatus*.

On day 13 of treatment, fibroplasia (increased number of fibroblasts/myofibroblasts) is evident in all the samples: within the fibrous granulation tissue most of the vessels disappears, and collagen is laid down and remodeled in an orderly pattern. Saline control maintains more blood vessels than *G. lucidum* and *P. ostreatus* mycelia, denouncing a slightly delayed remodeling process. The epidermis is completely restored when treated with both mycelia, while the tissue treated with saline shows a large part of the wounded area still without the epidermal layer. Moreover, PSR staining shows in all samples a large number of colored fibers, and only a narrow band of the wounded tissue appears collagen-free. A few fibers show orange to red birefringence in the zone with mature thick collagen fibers accumulation.

On the 18th day, the tissues treated with *G. lucidum* and *P. ostreatus* samples are characterized by an almost completely remodeled collagen, oriented in an appropriate orientation to withstand the tensile stresses placed on the area of repair. On the contrary, in 3 out of the 4 controls treated with saline solution large areas of collagen are still not completely remodeled, and many rounded and metabolically active fibroblasts are detected. In all cases, the epidermis is completely restored: both mycelia determine an epidermal layer fully restored in multiple layers of cells and with a fair degree of keratinization. Skin appendages such as hair follicles and glands reappear. On the other hand, the epidermal layer is still thin and not yet keratinized in the saline controls, highlighting a delay in the full lesion recovery.

PSR stain shows a continuous collagen layer, rich in orange-to-red fibers in both mycelia, while saline solution control shows large areas lacking organized collagen (only a few green fibers) within the dermal layer.

Moreover, the mycelia wettability was assessed in a previous work of ours and *P. ostreatus* demonstrates a hydrophilicity greater than *G. lucidum* mycelium. Since the mycelia are mainly based on not-soluble and not-swellable components as chitin, lignin, lipids and glycoproteins it is conceivable that no swelling occurs upon hydration and thus the water vapor permeability is not affected by the hydration. The hyphae entanglement is stable and presents interconnected pores to allow the exchanges of nutrients and the removal cellular metabolic products. This allows to have a scaffold able to sustain wound healing without change its 3D organization and microstructure (Antinori et al., 2021).

## Conclusion

*G. lucidum* and *P. ostreatus* mycelia are characterized by a porous structure made of microfibers resembling the network of polysaccharides and proteins typical of the dermal extracellular matrix, and can withstand sterilization by heating treatment, as observed via thermal analysis. Both mycelia are biocompatible, and *P. ostreatus* mycelium is characterized by the best performance. Both materials are able to enhance the collagen I gene expression, even though and *G. lucidum* performs slightly better. The β-glucans in the mycelia play a crucial role in promoting the synthesis of types I and III collagen, enhancing wound healing. These findings are confirmed by the wound-healing properties. Despite further studies are mandatory to fully characterize the mechanisms governing the cell- and tissue-mycelia interactions, to the best of our knowledge *in vivo* investigations constitutes a first milestone to prove that both mycelia are very promising candidates as green biomaterials to restore skin integrity in chronic skin lesions. As final remarks, *G. lucidum* and *P. ostreatus* mycelia are sustainable biomaterials, that perfectly resemble the ECM, paving the way of innovative biomaterials design.

## Data Availability

The raw data supporting the conclusion of this article will be made available by the authors, without undue reservation.
